# Understanding Cybersecurity Threat Trends Through Dynamic Topic Modeling

**DOI:** 10.3389/fdata.2021.601529

**Published:** 2021-06-29

**Authors:** Jennifer Sleeman, Tim Finin, Milton Halem

**Affiliations:** Department of Computer Science and Electrical Engineering, University of Maryland, Baltimore County, Baltimore, MD, United States

**Keywords:** cybersecurity, cyberthreat information, knowledge graph, topic modeling, dynamic topic modeling

## Abstract

Cybersecurity threats continue to increase and are impacting almost all aspects of modern life. Being aware of how vulnerabilities and their exploits are changing gives helpful insights into combating new threats. Applying dynamic topic modeling to a time-stamped cybersecurity document collection shows how the significance and details of concepts found in them are evolving. We correlate two different temporal corpora, one with reports about specific exploits and the other with research-oriented papers on cybersecurity vulnerabilities and threats. We represent the documents, concepts, and dynamic topic modeling data in a semantic knowledge graph to support integration, inference, and discovery. A critical insight into discovering knowledge through topic modeling is seeding the knowledge graph with domain concepts to guide the modeling process. We use Wikipedia concepts to provide a basis for performing concept phrase extraction and show how using those phrases improves the quality of the topic models. Researchers can query the resulting knowledge graph to reveal important relations and trends. This work is novel because it uses topics as a bridge to relate documents across corpora over time.

## 1 Introduction

Cybersecurity is a crucial computing area vital to our society due to the rise in cyberattacks and the damage they can do (Symantec, 2019). The risks are varied and include data breaches, service interruption, ransom demands, changing data, damaging physical infrastructure, election fraud, and introducing spyware to attack privacy. As our use of communication and networking technologies evolves, new vulnerabilities that had not been anticipated arise. For example, the increased use of machine learning and data analytics has led to risks of subverting artificial intelligence systems by “poisoning” their training data with biased or false information. It is essential to understand how cybersecurity vulnerabilities, risks, threats, and concepts are changing to keep our systems safe.

Researchers have used artificial intelligence techniques to extract information from documents such as security bulletins, after-action reports, and descriptions of new software vulnerabilities for many years. Most works in this area have used language understanding technology that extracts references to entities, such as malware instances, software products, IP addresses or process names, and relations between them. Although these data have been helpful for many purposes, they have not addressed temporal aspects of how the cybersecurity landscape has changed over the years. As computer exploits grow in sophistication, understanding past exploits and their evolution could provide insight into new exploits before they occur. A temporal analysis of cybersecurity documents may detect completely novel attacks, such as “poisoning” a machine learning system’s training data, soon after being anticipated or observed.

A work by Mittal et al. (2016) described the importance of the temporal dimension in understanding cybersecurity exploits, such as a 2015 attack against GitHub that lasted 72 h (Nestor, 2015). Often, it is not until the attack occurs that the information that led to it can be analyzed. However, in many cases, the attacker makes multiple attempts to execute the attack or spends time on a victim’s system before detection (Narayanan et al., 2018).

Norris et al. (2018), Norris et al. (2020) documented the increase in problems that local governments have experienced from various kinds of cybersecurity attacks, including phishing, data breaches, and ransomware. A trend of increasing attacks is evident, and, in several cases, they have virtually shut down city governments for significant periods. Noticing and documenting such trends within a community of targets will help researchers and public policy experts address the problem.

Applying a temporal analysis to cybersecurity language understanding can be done by applying dynamic topic models (DTMs) to documents that span a period of time. We show how to do this using two cyber-related document collections: 17 years of malware reports from Symantec and 20 years of cybersecurity research papers from the arXiv repository. We identify changes in trends over time for each and correlate the two models to show how new malware instances and behavior described in the first collection trigger and influence research changes in the second collection.

Domain-specific terminology, meanings, and jargon found in a narrow field like cybersecurity make concept spotting, information extraction, and natural language understanding difficult. The heavy use of acronyms and multi-word phrases with non-compositional semantics exacerbates the problem. To address these issues, we extract common cybersecurity concepts from Wikipedia data and identify phrases and acronyms that refer to them.

For the cybersecurity domain, applying and using dynamic topic models is still an unexplored area of research. Using DTMs and performing cross-domain analysis between multiple data sets extracted from cybersecurity collections are also a novel contribution. We put forth this work to show how temporal analysis by means of cross-domain understanding can be applied to cybersecurity and could be used to foster a document-based search tool.

## 2 Background

As the Internet and its use in everyday tasks has become ubiquitous, so has cybersecurity-related crimes. Cybersecurity attacks can be divided into those involving software, hardware, and networks (Jang-Jaccard and Nepal, 2014), and can attempt to exploit any combination of confidentiality, integrity, and availability (Goodman and Lin, 2007). Common attacks include the following from the study by Bendovschi (2015):

• Man in the middle attacks that intercept communication
• Brute force attacks that obtain and exfiltrate protected information
• Denial of service attacks that flood networks or services to lessen and prevent access
• Phishing attacks that use deception to fool people into disclosing sensitive information
• Social engineering attacks that manipulate users to obtain access to information
• Malware attacks that compromise data or resource integrity, confidentiality, and/or availability

### 2.1 Dynamic Topic Models

Dynamic topic modeling (DTM) (Blei and Lafferty, 2006) provides a means for performing topic modeling over time. Internally using Latent Dirichlet Allocation (LDA) (Blei et al., 2003), it creates a topic per time slice. By applying a state-space model, DTM links topic and topic proportions across models to “evolve” the models over time. Blei’s early work modeled the evolution of an extensive collection of articles from SCIENCE and showed topic evolution for specific scientific concepts of interest consistent with historical understanding. DTM has been used in many applications, including science research (Blei and Lafferty, 2006), software (Hu et al., 2015), finance (Morimoto and Kawasaki, 2017), music (Shalit et al., 2013), and climate change (Sleeman et al., 2016; Sleeman et al., 2017) to understand how particular domains have changed over time. We reported on the results of some initial early works in the cybersecurity domain in Sleeman et al. (2020).

## 3 Related Work

Research by Joshi et al. (2013) used information from cyber-based attacks and exploits to process unstructured text and to generate RDF-linked data that could then be used for identifying vulnerabilities. Although there are similarities to our approach, that is, converting unstructured text to a graph-based representation, our method treats documents as mixture models enabling improved similarity detection among documents. Also, our method includes temporal analysis of documents. Mathews et al. (2016), Mathews (2017) built on this work to develop a complete intrusion detection system that used machine learning but addresses different issues.

More recent research by Kang et al. (2016), Prakash (2016) has an approach similar to ours; their method is generative. They use propagation-based models to represent malware trends by using phrases that provide contextual constraints to help identify malware attacks. Their goal differs from ours, which uses models as a way of gathering information about historical events and current research that could then be used to support systems that do such predictions.

Kolini and Janczewski (2017) used topic modeling for processing national cybersecurity strategies (NCS) documents in addition to hierarchical clustering. They used the topics as a means for finding the themes among the NCS documents. Their topic analysis includes using human annotators. Our work differs in that we are evaluating how concepts are changing over time by means of a dynamic topic model.

## 4 Approach

We use dynamic topic models (DTMs) to evolve topics over time in data collection. A key innovation to our method is using Wikipedia concepts to provide domain context for preprocessing the documents. Typically, a bag-of-words approach is used for methods such as topic modeling. However, in domains with technical terminology, we have found that using concepts that include domain-relevant phrases and explicitly looking for those concepts during document preprocessing improves topic modeling results (Sleeman, 2017; Sleeman et al., 2018).

Automatically extracting domain concepts from a text collection is a challenging problem. An important contribution of this work is our approach to automatic domain concept extraction using Wikipedia concepts. In our previous work (Sleeman et al., 2018), we showed how to use document repository glossaries to extract domain concepts and how these domain concepts could then be harnessed to extract phrases from documents for topic modeling processing. We showed that typical bigram and trigram extractors underperform in finding domain concepts (recovered 6% of the concepts for the climate change use case) compared to the concepts extracted from domain glossaries.

**FIGURE 1:**
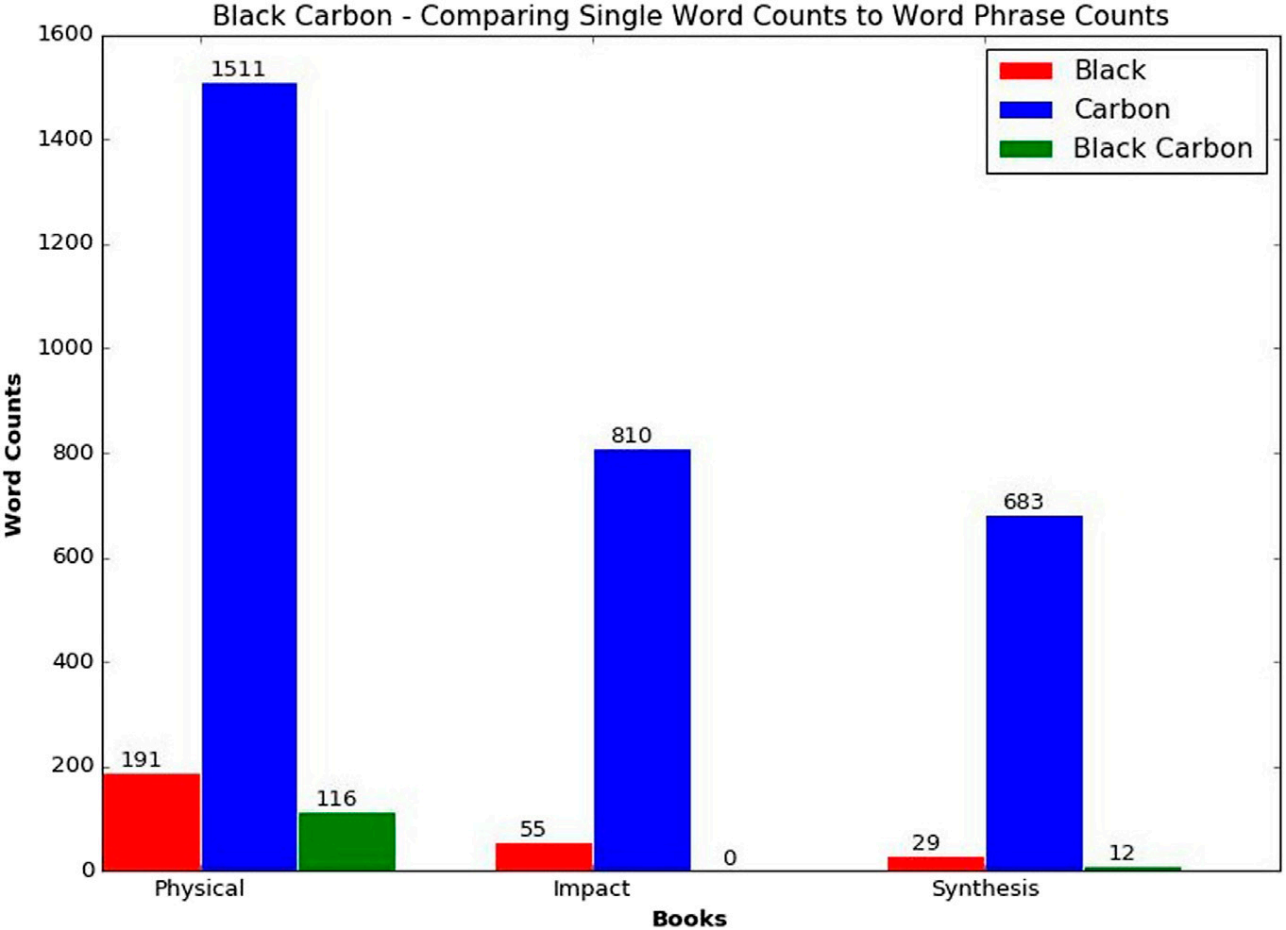
Comparing word counts and phrase counts for black carbon among the intergovernmental panel for climate change books assessment report 3 (Sleeman et al., 2018).

For scientific data and domains with domain-specific jargon, starting with known concepts provides a means for building a suitable set of phrases to use during extraction. As we showed in our previous work and highlight in Figure 1, processing these known concepts as phrases instead of individual words enhances the vocabulary, allowing us to generate more intuitive topics.

In our current work, we build on this previous approach by using the Wikipedia concept hierarchy to find domain-specific concepts. We exploit Wikipedia concepts related to cybersecurity as a context model for training the DTM. Since Wikipedia concepts are easily mapped to concepts in DBpedia (Auer et al., 2007), Wikidata (Song et al., 2020), and other background knowledge resources, we use knowledge graphs during the modeling process, enabling the results of this work to be used for additional query and inference.

With this context in place, we preemptively search for mentions of cybersecurity concepts in the text of each document before standard text processing methods are used, such as stop word removal, low-frequency term removal, and lexical-based processing. In addition to the Wikipedia-based concept search, we apply standard text processing to find other words of interest subject to stop word removal, low-frequency removal, and lexical-based processing.

**FIGURE 2:**
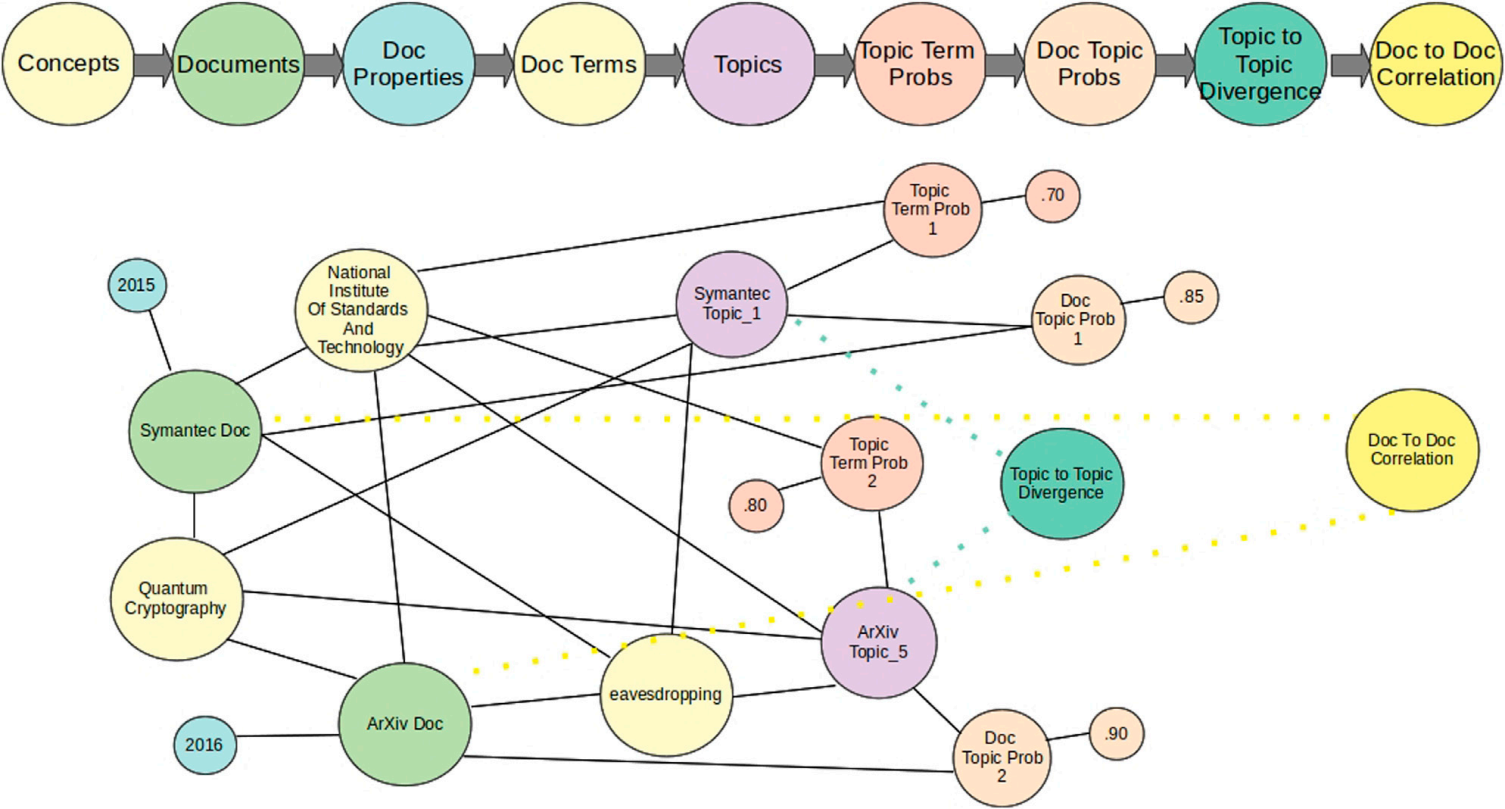
Knowledge graph construction of the dynamic topic modeling process.

We generate three files from the collection: one large vocabulary file across all time slices, a file that defines the words for each document, and a file that indicates to the dynamic topic modelling system how many files exist per time slice. We then use the dynamic topic modelling system to generate the topic model. The whole process is governed by a knowledge graph created and updated as documents in the repository are processed. The knowledge graph is then populated as the dynamic topic models learns latent topics over time, as shown in Figure 2.

Initially, the knowledge graph encapsulates the concepts from Wikipedia for the specific domain. As more documents are processed, it includes graphs of the documents in the collection(s) and their properties, including their discovered concepts. We add graphs of the topics and topic probabilities to the knowledge graph during the topic modeling process. For cybersecurity, this is particularly useful when looking for documents with common exploit properties.

### 4.1 Extracting Knowledge From Unstructured Text

We captured a total of 3,836 concepts from Wikipedia which were used to establish the context for the topic modeling portion. We started with the concept phrases “cybersecurity,” “computer security,” “cyber security,” and “cyber.” For each phrase, we retrieved Wikipedia pages; then for each page retrieved, the outgoing links were found on that page. We perform a one-level traversal to formulate the concept list. The longer this list of concepts, the longer is the preprocessing time. When we increased the traversal to three levels, processing time doubled. Some example concepts are shown in Table 1.

**TABLE 1:** Examples of Wikipedia concept terms used.

*Example Concept*
Cryptanalysis
Cryptographic protocol
Cryptographic software
Cryptography
Cryptosystem and cryptovirology
Cyber-insurance
Cyber-security regulation
Cyber security standards
Cyber self-defense
Cyberattack and cybercrime
Cyberspace
Cyberterrorism
Cyberwarfare

When we perform the preprocessing step, which is the step that finds the word and word phrases in the text and accumulates their frequencies, we then treat the acronym for a word phrase as if that phrase was found and increase its frequency count. The task of automatically generating possible acronym is difficult because acronyms can be confused with stop words and can also be polysemous. For example, a simple Wikidata query shows that 32 entities have an English name or alias matching “CPA.”

We use a general heuristic to extract acronyms from phrases that takes the first letter of each word in the phrase (excluding pronouns) and generates an acronym by concatenating the letters. We captured 245 cybersecurity acronyms based on the cybersecurity Wikipedia concepts. Some example acronyms based on our concepts are shown in Table 2. From this list, one can see that our heuristic for choosing acronyms works well for finding common acronyms. More complex acronyms, however, are harder to be identified with a heuristic. In some cases, additional acronyms could be found by matching the concept to a Wikidata entity and identifying acronym-like strings among its aliases.

**TABLE 2:** Examples of acronyms generated for cybersecurity-related concepts.

*Example Concept*	*Acronym*
Australian information security association	aisa
Advanced encryption standard	aes
Denial of service	dos
Department of homeland security	Dhs

In particular, for the cybersecurity domain, another issue that commonly arises relates to variations of phrases. For example, *“W97M.Coldape macro virus*” is also seen as *“W97M.Coldape virus”* and *“W97M.Coldape*.” When calculating word frequencies, treating these as the same concept is important for statistical significance. Our topic model ontology, described in more detail in Section 4.3, supports alternative names in addition to acronym support.

### 4.2 Topic Models Over Time

In this work, we used dynamic topic models to model multiple document collections over time. The output from preprocessing is a set of files that are required by DTM including the vocabulary file representing the vocabulary for the document collection, a file which indicates the number of time slices, and a file that defines the words and frequencies for each document. We then apply our cross-domain topic modeling methodology to find topic pairs that have low divergence scores based on Jensen–Shannon divergence (Lin, 1991).

For each topic in domain 1 described by $d_{1}$ and each topic in domain 2 described by $d_{2}$, using the term probabilities in each topic, we take the *n* most probable terms and generate a new vector of term *V* that consists of the top terms from $t_{d_{1}}$ and the top terms from $t_{d_{2}}$. For each term in *V*, if the term exists in $t_{d_{1}}$, we assign the probability from $t_{d_{1}}$ to $V_{t_{d_{1}}}$. If the term exists in $t_{d_{2}}$, we assign the probability from $t_{d_{2}}$ to $V_{t_{d_{2}}}$. We normalize $V_{t_{d_{1}}}$ and $V_{t_{d_{2}}}$ such that their probabilities are redistributed and sum to one. This results in two new probability distributions for $t_{d_{1}}$ and $t_{d_{2}}$.

After we perform the above process for every pair of topics across $d_{1}$ and $d_{2}$, we find the Jensen–Shannon divergence pairs with divergences below a given threshold. The Jensen–Shannon divergence between two probability distributions P1 and P2 is defined as

$$JSD\left[P1,P2\right]=\frac{1}{2}\left(KL\left[P1,\frac{P1+P2}{2}\right]+KL\left[P2,\frac{P1+P2}{2}\right]\right),$$ (1)

where KL is the Kullback–Leibler divergence.

The smaller the threshold, the fewer the pairs will be used to obtain documents across the two domains. Given a pair of topics below the divergence threshold, documents for each topic are discovered based on a second threshold, which defines how much the document mixture model should pertain to the paired topic. This method can be compared to an unsupervised clustering technique in that documents are grouped together based on two metrics: one measuring how much the topic contributes to the document’s mixture and the other measuring how low the divergence is between any given topic pairs. We use this method to map unknown documents to a known set of documents to understand the unknown document better.

### 4.3 Automatic Knowledge Graph Generation and Use

The further contribution of this work is the topic model ontology (TMO) (Sleeman, 2020) which provides a means for structuring knowledge that can be used for both end user discovery and as an input into downstream classification methods. By generating knowledge graphs that represent the documents, their words and frequencies, the topics and topic pairs, and by grounding all of this by the Wikipedia concepts, the knowledge acquired from this processing can be used for other applications such as search and discovery. By linking the predefined concepts to Wikipedia-based concepts, automatic Wikipedia page linking is realized.

**FIGURE 3:**
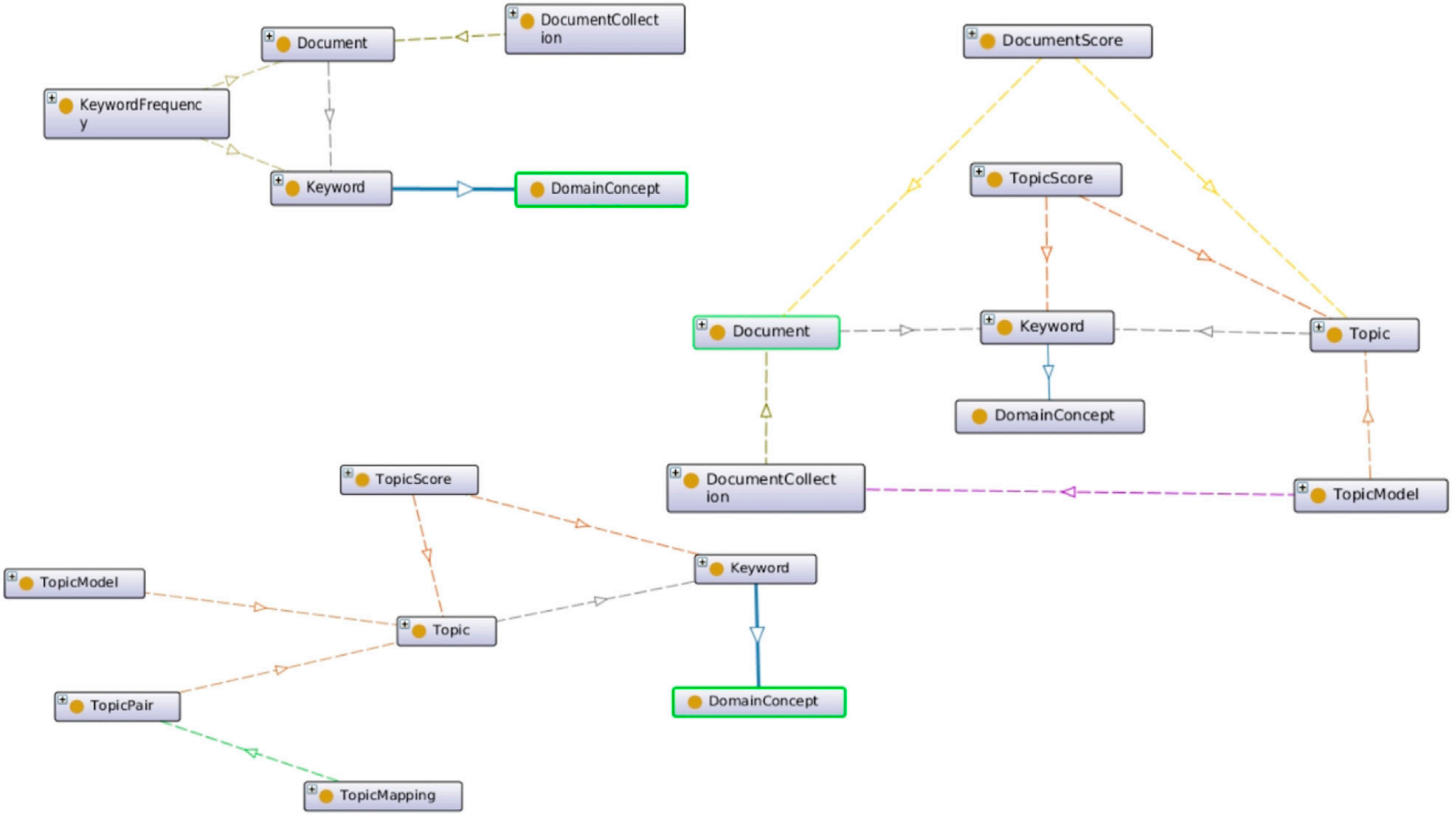
Three components of our topic model ontology (TMO) are **(A)** classes for representing document collections **(top left)**, **(B)** classes to encode document and topic relationships **(middle right)**, and **(C)** classes to represent cross-domain mappings **(bottom left)**.

Based on our early work (Sleeman, 2017; Sleeman et al., 2018; Sleeman et al., 2020), we further developed and extended a custom OWL ontology that we created for automatic topic model knowledge graph generation. The schema represents document collections in terms of domain concept phrases. When a document is parsed, an instance of that document is represented in the knowledge graph by the keywords and concepts found in it, as shown in Figure 3.

We also capture relationships between documents and topics while generating the topic model. In our knowledge graph, we use the TMO schema to represent each topic and its top *N* terms, which map back to keywords and concepts captured when processing each document, as shown in graph fragment in the upper left corner of Figure 3.

In addition, as we perform cross-domain correlations, we are able to represent topic mappings ontologically, as shown in the graph in the bottom left portion of Figure 3. For example, when we look for cross-domain mappings between the arXiv data collection and the Symantec data collection, we can represent topics that correlate to each other by means of topic mappings. These connect back to their respective topics, which in turn are linked to the keywords and concepts that are linked to the documents processed.

## 5 Cybersecurity Data Sets

We evaluated two different data sets over time for this work. The first is a collection of research papers from arXiv categorized by their authors as relevant to Cryptography and Security. Documents in this set tend, on average, to be relatively long. The second is a collection of Symantec Malware reports that are uncategorized. Their documents tend, on average, to be shorter. The number of arXiv papers increases steadily over time, and the number of Symantec Malware reports is strongly correlated to the frequency of security exploit events. We describe each of these data sets in more detail below.

**FIGURE 4:**
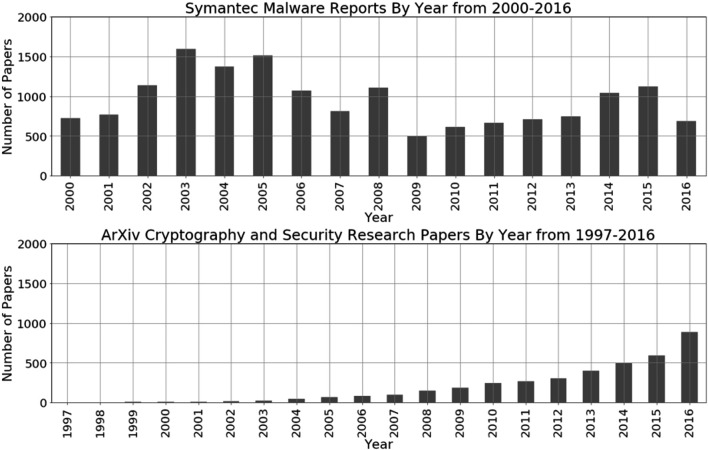
Symantec malware reports from 2000 to 2017 data distribution by year **(top)** and Cryptography and Security arXiv articles from 1997–2016 data distribution by year **(bottom)**.

The Symantec Malware reports data set is a set of reports which describe malware incidents and actions to take to combat the malware. They are typically short, with an average of about 9 KB size or approximately 4,500 words. There are a total of 16,167 files with a total size of approximately 149.6 MB. This data set spans 17 years, from 2000 to 2016. These data are unlabeled. However, in each report, there is a text that indicates the *type* of the report with the types “trojan,” “worm,” and “virus” as the highest types present across reports. We show the distribution by year in Figure 4.

The arXiv Cryptography and Security data set is a set of research papers related to cryptography and security. They are typically longer in size, with an average of about 50 KB size or approximately 20,000 words. There are a total of 3,913 files with a total size of approximately 215.5 MB. The documents are categorized as “Cryptography and Security,” but in addition to this base category, many are further tagged by the other categories, with about 94 categories in total. This data set spans 20 years from 1997 to 2016, as shown in Figure 4.

## 6 Experiments and Analysis

To assess the value of our approach, we performed two different experiments. One experiment assesses the improvement in the quality of the topics using concept phrases which are obtained from Wikipedia. A second experiment measures how the dynamic topics can be used in understanding how the cybersecurity domain is changing over time.

### 6.1 Concept Context Experiment

In this experiment, we tested how the topic model performs given the Wikipedia-provided concepts as the context for the models in comparison with a standard bag-of-words model without a predefined context. We used the arXiv data set, labeled by category. We split the data set into a train and test set (60/40 split). We then built two separate topic models, one which has the concept-provided context and the second which does not use a context but rather a standard bag of words. We performed the similarity portion of the experiment without the temporal component in order to isolate the concept context portion of this work strictly. We evaluate document similarity by grouping documents by the subcategory type.

**FIGURE 5:**
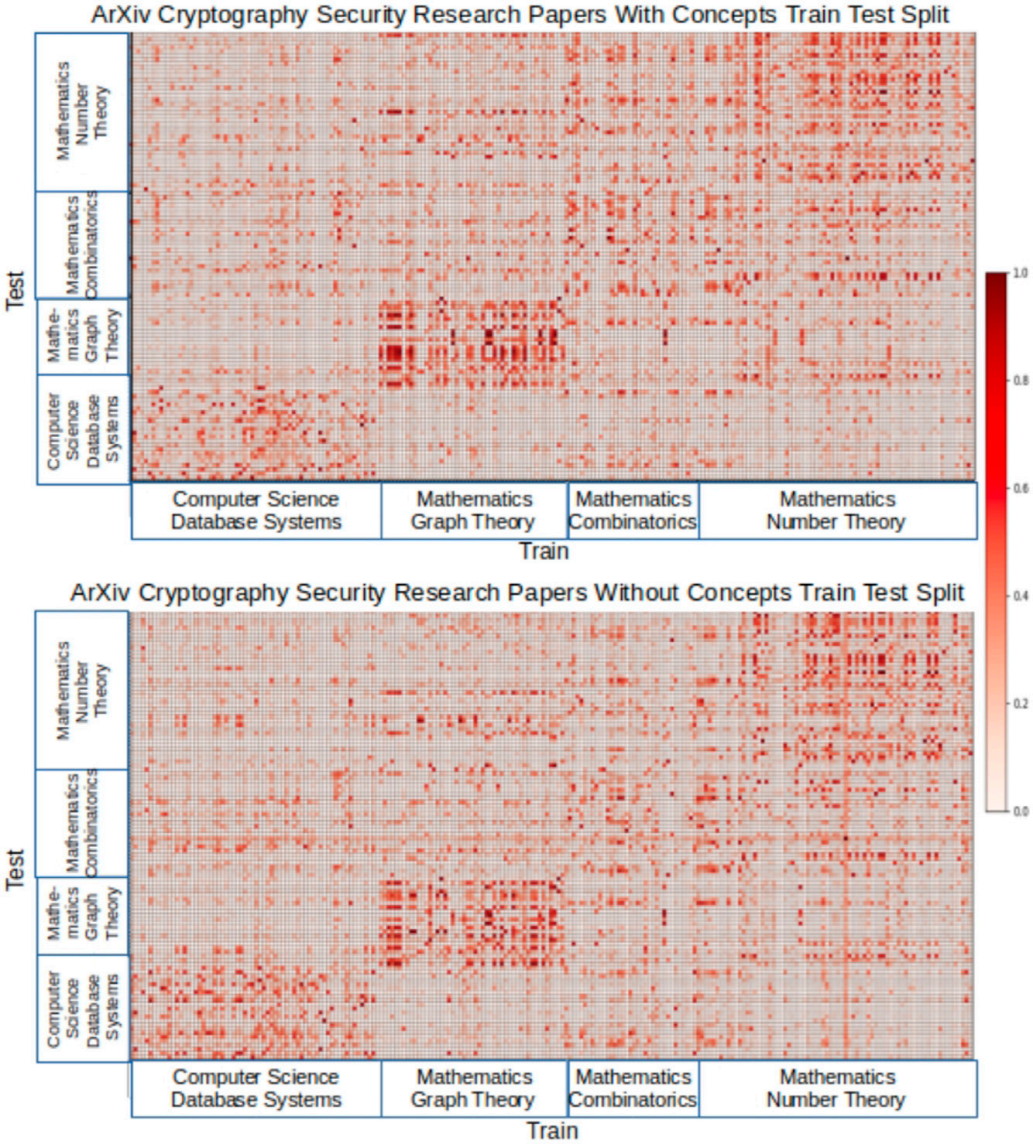
arXiv train/test split heat maps.

We generated topic models for the arXiv data set with and without the concept context using the training set and then used the test set for evaluation. We measured similarity defined by the average probabilities across documents for each topic. Figure 5 shows two heat maps comparing the arXiv topic models with and without concepts. For visualization, we show a subset of documents from the train and test sets, organized by their paper subcategories. The heat map shows subcategory paper similarity between the train and test documents, with darker cells indicating stronger similarity.

We can observe two things from the heat maps. For certain subcategories of papers, we see stronger similarity between train and test in the concept model, such as the mathematics graph theory train test section in the heat maps. Sometimes, this difference in similarity is subtle but still evident, as in the mathematics number theory train test section in the heat maps. Small differences can indicate significant improvement in topic word probabilities that are correlated to a given set of documents. As we will show in Table 3, the top ten topic word probabilities tend to be more human understandable with concept context models. Second, the heat maps show that with the concept model, there were fewer incidents of test documents with similarities that were the same across all subcategories, as indicated by a horizontal or vertical band. For those documents, the concept model was better at distinguishing between subcategories.

**TABLE 3:** These two examples of the top ten terms for a topic from the arXiv topic model of the DTM concepts with and without context concept phrases show that adding the phrases makes the concepts more easily understood by a person.

*Year*	*Topic With Concept Phrases*	*Topic Without Concept Phrases*
2000	Quantum cryptography, phase, photon, cryptography, measurement, channel, system, eavesdropping, stage, and polarization	Quantum, state, communication, phase, cryptography, channel, eavesdropping, protocol, error, and polarization
2000	Intrusion detection, universal, taxonomy, intrusion detection system, based, payload, classification, input, attack, and alert	Cell, network, intrusion, parameter, system, information, detection, method, space, and approach

When comparing the dynamic topic modelling using 100 topics and the full data sets, the first difference we observed is the vocabulary size. With the concept model, the size of the vocabulary is 13,016, and the vocabulary size for the non-concept model is 11,824. Between the two models, as presented in the first example in Table 3, there are two real differences. In the concept model, topics tend to contain word phrases based on the Wikipedia-built concept model. This affects the topics because when a phrase is broken into a sequence of words, other interesting words become less probable. For example, *quantum cryptography* was a concept defined by our knowledge graph. The word *photon* is seen in the top ten most probable words, but it is not in the top ten most likely words for the topic without context concepts.

The second difference is since the model is guided by the domain concepts, words that are harder to interpret because they are more generic move farther toward the tails of the probability distribution. In the second example in Table 3, *intrusion detection* is a concept specific to the domain and found in the text. In the topic model without concepts, *intrusion detection* is never found. The only topic that mentions *intrusion* and *detection* contains words that are more generic and less specific to the domain.

### 6.2 Contextual Classification Experiments

**FIGURE 6:**
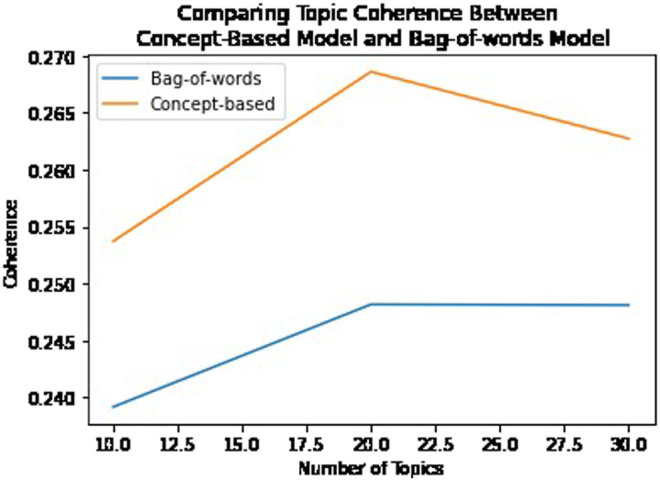
Comparing topic coherence between the bag-of-words model and the concept-based model using the c_v measure.

Although the approach we describe is meant to improve human topic understanding, we also evaluated how the cybersecurity domain concepts affected classification performance by running a classification experiment using the concept-based method and compared this with a bag-of-words method and an automatic phrase extraction method called Phrasemachine Handler et al. (2016). We processed 2,400 documents from the Cryptography and Security data set across 17 classes equally distributed. We performed a randomized train test split (70–30) and applied lemmatization, stop word removal, and other preprocessing techniques. All of these techniques were applied in the same manner to each method. We evaluated the output of three different classifiers (SVM, naive Bayes, and logistic regression) for each method. To enable the processed documents to be used by the classifier, we applied a TF-IDF vectorization. However, to ensure the method was fairly evaluated, we do not set a maximum number of features nor do we apply a second stop word removal during vectorization. The results are shown in Table 4. Again, the effects of the concept-based method can be subtle; however, this experiment shows that when a state-of-the-art phrase extraction method is used, it will unfortunately include many noisy phrases that are not domain concepts. Whereas the bag-of-words approach results in similar classification accuracy when compared with the concept-based approach, the topic model coherence shows the concept-based model is more coherent using the c_v Syed and Spruit (2017) measure in Figure 6.

**TABLE 4:** Comparing the concept-based, bag-of-words, and automatic phrase extraction methods for a classification task using three different algorithms on the Cryptography and Security data set.

Classification method	*Concept-Based Accuracy*	*Bag-of-Words Accuracy*	*Phrasemachine Accuracy*
SVM	0.60	0.59	0.58
Naive bayes	0.36	0.34	0.28
Logistic regression	0.59	0.59	0.56

### 6.3 Dynamic Model Experiment

In this experiment, we measured how reliable the model is for understanding specific concept evolution. We build the dynamic topic model for the arXiv data set and the Symantec Malware data set. We compare topic evolution for a range of 20–100 topics using intervals of 20. We also experimented with the variance hyperparameter in the DTM which controls how much time variance is allowed among topics; however, we used a low variance of 0.05.

When we build topic models over time, topics evolve over time based on the documents in the collection at that time point. Our observation is as we increased the number of topics, we saw more granularity among topics. Topics represented more narrow mixtures. We also observed concepts that drop off of one topic and fall into another at various points in time. Deciding on the number of topics can be based on a measure called perplexity, which approximately gives a good estimate. Another approach is to observe how the topics change visually as the number of topics increases and choosing the total number of topics based on the requirements of the problem being solved. For cybersecurity, we suggest using a larger number of topics for more granularity. Since discovery is a big part of the cybersecurity process, that is, looking for information based on past exploits, the more granular the model, the more information can be inferred. However, we have found that since concepts can be represented in different topics simultaneously over time due to the co-occurrence of the concept with other words, plotting the concept over time across all topics provides a visual trend of how the concept is changing over time.

**FIGURE 7:**
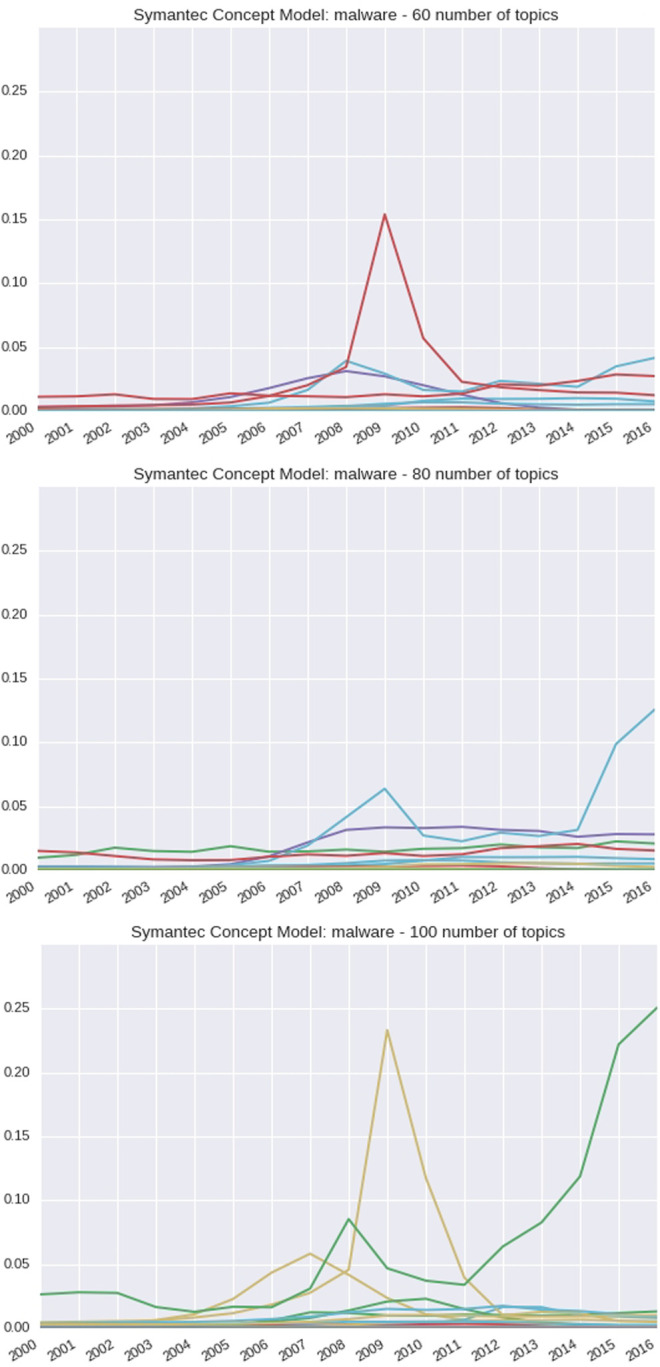
60-, 80-, and 100-topic Symantec Malware reports of the DTM concept: Malware.

Using the concept *malware* as a use case and observing all topics for a given model, Figure 7 shows how the probability for the word malware changes over time. As the number of topics is increased, the significant spike at 2009 remains prominent across models for a given topic. In addition, there is a second trend that is increasing as it approaches 2016, which is more prominent as the number of topics is increased.

According to Wikipedia, the first ever *malware* was detected on February 16, 2006 (Wikipedia Contributors, 2019). The models reflect a rise in probabilities from approximately 2005 to the spike in 2009; however, as we obtain more granular details using 100 topics, it is observed that the secondary trend dominates around 2011.

In Table 5, we show topics that correlate to the two trends that spike early and then drop off in Figure 7 100 topics. The model begins to capture the reference of *malware* in the top ten most probable terms for these two trends. The first trend which has a smaller spike starting around 2006 shows association of *malware* with how to protect computing devices using *antivirus software*. The second trend which has a more pronounced spike relates to mobile computing and *malware*. The *malware* presence in the top ten most probable terms is also observed in the models created with 60 topics and 80 topics.

**TABLE 5:** The top ten most relevant terms from two topics in the 100-topic Symantec Malware report of the dynamic topic model that correlate to the two trends that spike early and then drop off in Figure 7 100 topics.

*Year*	*Topic 5’s Top Ten Most Relevant Terms in Symantec Reports*
2006	Remove, protection, threat, antivirus software, packed, file, malware, Symantec, security, and window
2007	Remove, protection, threat, antivirus software, packed, file, malware, Symantec, security, and window
2008	Protection, remove, threat, malware, packed, file, Symantec, antivirus software, security, window, protection, packed, threat, file, Aymantec, remove, malware, window, antivirus software, and security
*Year*	*Topic 41s Top Ten Most Relevant Terms in Symantec Reports*
2008	Privacy, commander, doctor, malware, picture, movie, action, video, multi, and surveillance
2009	Malware, doctor, privacy, commander, action, android, intent, picture, movie, and video
2010	Malware, action, doctor, privacy, android, intent, commander, Bluetooth, picture, and provider
2011	Action, android, intent, privacy malware, doctor, Bluetooth, commander, picture, and WiFi

In the 100-topic model, the third trend observed has a moderate spike around 2008 but then steeply increases from 2011 onward and is shown in Table 6. This trend indicates a new class of attacks.

**TABLE 6:** 100-topic Symantec Malware report of the dynamic topic model topic 86.

*Year*	*Topic 86’s Top Ten Most Relevant Terms in Symantec Reports*
2007	Malicious, component, info, scanner, attacker, rootkit, door, remote, malware, and computer
2008	Info, malicious, malware, scanner, attacker, rootkit, component, door, computer, and remote
2009	Malicious, info, attacker, malware, door, scanner, rootkit, remote, computer, and component
2010	Malicious, info, attacker, malware, door, computer, remote, scanner, rootkit, and based
2011	Info, malicious, attacker, malware, computer, door, remote, reputation, dropped, and based
2012	Malicious, info, attacker, malware, computer, door, remote, reputation, threat, and dropper
2013	Malicious, info, malware, attacker, computer, remote, door, dropper, reputation, and back
2014	Malicious, malware, info, attacker, computer, dropper, remote, door, reputation, and back
2015	Malware, malicious, info, dropper, attacker, computer, remote, payload, dropped, and door
2016	Malware, malicious, info, dropper, attacker, computer, remote, payload, dropped, and door

Although the first official *malware* detection was in 2006, in the 100-topic model, as shown in Table 7, *malware* was in the top ten most probable words for a particular topic in 2000. Indeed, a Symantec Malware report did specifically reference *malware* with a time stamp of 2000. In fact, this report details an exploit titled *Infostealer* which was found on *December 8*, *1997*.

**TABLE 7:** 100-topic Symantec Malware report of the dynamic topic model topic 86.

*Year*	*Topic 86’s Top Ten Most Relevant Terms in Symantec Reports*
2000	Component, computer, attacker, malicious, dropper, kernel, remote, malware, door, and author
2001	Component, attacker, computer, malicious, remote, dropper, door, malware, kernel, and security
2002	Component, attacker, malicious, door, dropper, computer, remote, kernel, malware, and rootkit

**FIGURE 8:**
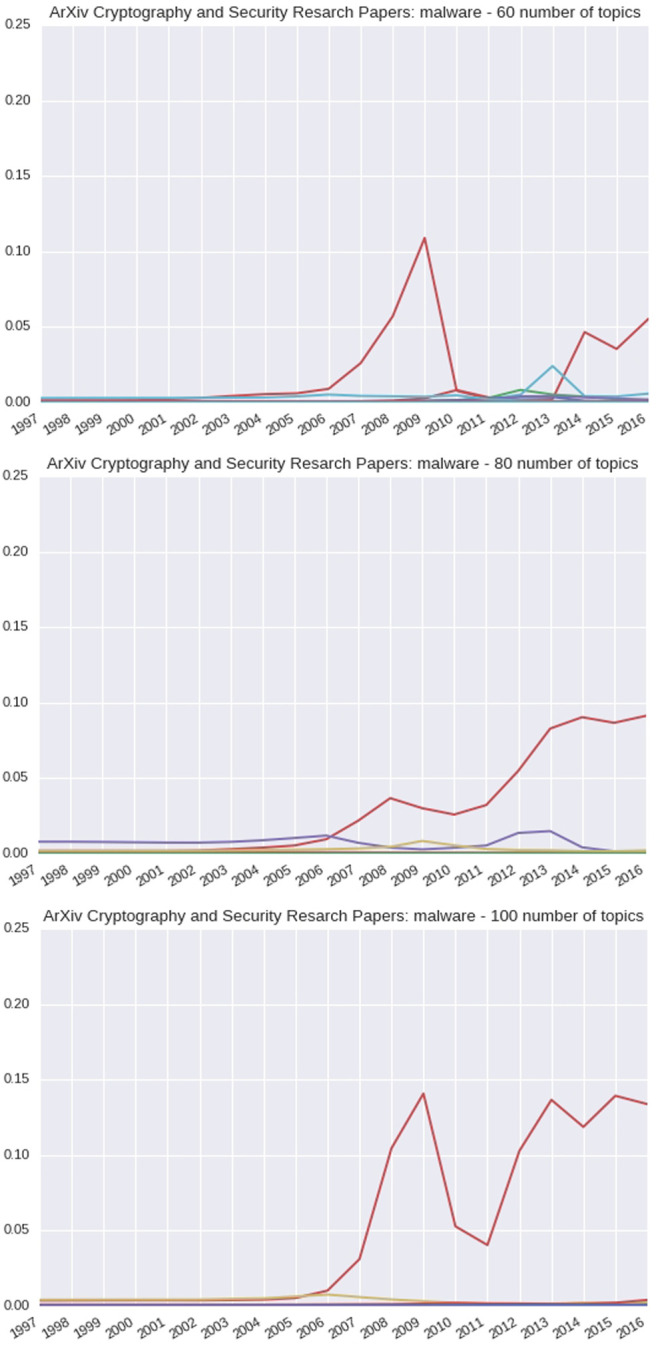
60-, 80-, and 100-topic arXiv Cryptography and Security research articles of the DTM concept: Malware.

When observing the same trend information for the arXiv Cryptography and Security research papers, as shown in Figure 8, there is a spike among one topic in particular at 2009 for 60 topics. This spike is more prominent at 2008, given 80 topics, and at 2009, given 100 topics. There appears to be a dip and then another rise as it reaches 2016 in each.

For the arXiv Cryptography and Security research papers, *malware* is found in the top ten probable words by 2007, as shown in the first table in Table 8. By 2012, for topic three, malware was the highest probable word as shown in the second table. By 2012, for topic three, malware was the most probable word.

**TABLE 8:** 100-topic arXiv Cryptography and Security research articles of the dynamic topic model topic 3 for years 2007–2009 and 2012–2016.

*Year*	*Topic 3’s Top Ten Most Relevant Terms in arXiv Articles, 2007–2009*
2007	Infected, epidemic, virus, malware, infection, worm, internet, and spreading
2008	Wireless, spread, malware, worm, infected, infection, spreading, and virus
2009	Spread, internet, propagation, epidemic, malware, worm, infected, infection, virus, propagation, host, Internet, simulation, and spread
*Year*	*Topic 3’s Top Ten Most Relevant Terms in arXiv Articles, 2012–2016*
2012	Malware, infection, infected, virus, worm, malicious, host, behavior, spread, and parameter
2013	Malware, malicious, behavior, worm, infected, sample, call, email, file, and family
2014	Malware, similarity, sample, infected, behavior, infection, malicious, family, based, and type
2015	Malware, sample, behavior, spreading, family, infected, based, malicious, virus, and benign
2016	Malware, sample, virus, family, malicious, benign, infected, infection, based, and anti

However, since the arXiv data set is a collection of research papers rather than a set of detailed exploit reports, the effects are less pronounced. Also, there tends to be a delay in concepts reflected in research papers due to the time to perform research and write the paper. The Symantec reports, in contrast, are typically focused on a single narrow vulnerability, exploit or attack, and of a more urgent nature.

Given this type of analysis, one could use these models to understand how long it takes to build momentum among published work for a particular attack type. We could potentially use this information to identify the time period that might have the most relevant work for a given attack type or to predict future attacks based on currently popular research concepts.

## 7 Using the Knowledge Graph for Search

Modeling the topic modeling process in support of cross-domain mapping provides the benefit of finding documents that are similar across domains through mapped topics. This could be invaluable for scientific domains and domains such as cybersecurity, where discovering new information relevant to cybersecurity concepts can yield information to help identify potential threats.

In the cross-domain case, where we built topic models for both the arXiv Cryptography and Security research papers and the Symantec reports, finding relevant research papers both before an attack and after an attack can help in the information discovery process. For example, a Symantec report dated 2007 included the following concepts: *adware*, *malware*, *microsoft*, and *windows_xp*. When we found documents that were correlated and shared concept occurrences, we discovered research papers that may be informative. A research paper published in 2004 entitled “Modelling the costs and benefits of Honeynets” has many concepts in common, including *malware*, *computer_crime*, *forensic*, *honeynet_project*, *keystroke_logging*, and *network_security*. This document could provide insight as to how to thwart a potential attack of this type. Another research paper published in 2016 with the title “Combating Malicious DNS Tunnel” described similar attacks and included the following concepts: *command_and_control*, *denial_of_service*, *fraud*, *malware*, *mcafee*, *national_institute_of_standards_and_technology*, and *theft*.

The discovery of related documents also applies to documents within a single domain. For example, after modeling documents from the arXiv Cryptography and Security research papers, we were able to find a document published in 2012 related to biometrics with the title “Secure voice based authentication for mobile devices: Vaulted Voice Verification.” We modeled this document in terms of known concepts that included *biometric*, *cryptosystem*, *information_security*, *mobile_device*, and *transport_layer_security*. This allowed us to recognize that it was similar to a later document published in 2016 with the title “Attacks on Fitness Trackers Revisited: A Case-Study of Unfit Firmware Security.” That document had many overlapping concepts, such as *information_security* and *transport_layer_security*.

## 8 Conclusion and Future Work

Cybersecurity threats are increasing. As systems evolve, new vulnerabilities are discovered because threats are also evolving and developing new attack strategies, leading to the creation of new categories of attacks. Tools to help model and understand such trends in cybersecurity threats and attacks are useful in helping combat them. In this work, we have provided insight into how to use dynamic topic models for cybersecurity documents to understand how the concepts found among documents are changing over time. We demonstrated an approach that uses an ontology of cybersecurity concepts extracted from Wikipedia to find phrases that improve the readability of the topics and provide better human understanding of the topics. We represent the results of the dynamic topic model as a knowledge graph that can also be used for inference and information discovery.

Our future work will include automatic concept acquisition using phrase extraction methods. As the knowledge graph is built over time, we will apply a method for automatically identifying new concepts that are representative of the domain. Our preliminary work on this shows that it can improve the models and produce better results. We also plan to build a user interface to support knowledge search and discovery over a learned topic model knowledge graph. This capability will enable domain experts to run searches against the knowledge graph, discover new knowledge, and serve as active components in human-in-the-loop architectures.

## Data Availability

The ontology described in this paper is available at https://github.com/Ebiquity/topic_model_ontology and can be used in RDF datasets as http://purl.org/tmon.
